# Development of a Biosensor for Detection of Benzoic Acid Derivatives in *Saccharomyces cerevisiae*

**DOI:** 10.3389/fbioe.2019.00372

**Published:** 2020-01-07

**Authors:** Sara Castaño-Cerezo, Mathieu Fournié, Philippe Urban, Jean-Loup Faulon, Gilles Truan

**Affiliations:** ^1^TBI, Université de Toulouse, CNRS, INRA, INSA, Toulouse, France; ^2^Micalis Institute, INRA, AgroParisTech, Université Paris-Saclay, Jouy-en-Josas, France; ^3^Chemistry School, Manchester Institute of Biotechnology, The University of Manchester, Manchester, United Kingdom

**Keywords:** *p*-hydroxybenzoic acid, *p*-aminobenzoic acid, biosensor, synthetic biology, yeast

## Abstract

4-hydroxybenzoic acid (pHBA) is an important industrial precursor of muconic acid and liquid crystal polymers whose production is based on the petrochemical industry. In order to decrease our dependency on fossil fuels and improve sustainability, microbial engineering is a particularly appealing approach for replacing traditional chemical techniques. The optimization of microbial strains, however, is still highly constrained by the screening stage. Biosensors have helped to alleviate this problem by decreasing the screening time as well as enabling higher throughput. In this paper, we constructed a synthetic biosensor, named sBAD, consisting of a fusion of the pHBA-binding domain of HbaR from *R. palustris*, the LexA DNA binding domain at the N-terminus and the transactivation domain B112 at the C-terminus. The response of sBAD was tested in the presence of different benzoic acid derivatives, with cell fluorescence output measured by flow cytometry. The biosensor was found to be activated by the external addition of pHBA in the culture medium, in addition to other carboxylic acids including *p*-aminobenzoic acid (pABA), salicylic acid, anthranilic acid, aspirin, and benzoic acid. Furthermore, we were able to show that this biosensor could detect the *in vivo* production of pHBA in a genetically modified yeast strain. A good linearity was observed between the biosensor fluorescence and pHBA concentration. Thus, this biosensor would be well-suited as a high throughput screening tool to produce, via metabolic engineering, benzoic acid derivatives.

## Introduction

Synthetic biology proposes cutting-edge methodologies for using natural resources (Jullesson et al., 2015; Smanski et al., 2016; Le Feuvre and Scrutton, 2018; Schindler et al., 2018), understanding basic cellular functions (Metzger et al., 2018; Toda et al., 2018) or reprogramming cell fate (Black and Gersbach, 2018). Biosynthesis of small organic molecules by microorganisms, instead of manufacturing them from petroleum-sourced synthesized chemicals, is one of the main objectives of metabolic engineering (Khalil and Collins, 2010; Markham and Alper, 2015; Li et al., 2018). While the demand for green (sustainable) chemistry is growing steadily, there are still few examples of successful industrial production of molecules from microorganisms. The clear bottleneck in metabolic engineering lays in the low throughput of analytical techniques used to determine products yields compared to the rate of the construction of new engineered microorganisms (Rogers and Church, 2016). To overcome this limitation, natural or synthetic biosensors are potential tools capable of correlating the concentration of a target chemical within the cell to an easily monitored output signal, such as fluorescence (Farmer and Liao, 2000; Schulman and Heyman, 2004; Dietrich et al., 2010; Liu et al., 2017; Mannan et al., 2017).

The yeast *Saccharomyces cerevisiae* is a widely used model organism and production host, due to a comprehensive collection of metabolic engineering tools and deep knowledge of its genetics accumulated over half a century. However, the development of synthetic biosensors in yeast somewhat lags behind those available for *E. coli* (Leavitt and Alper, 2015). One of the first transcription factor-based biosensors was exemplified with the NhaR system from *Pseudomonas putida*. This system could drive the transcription of β-galactosidase as a function of benzoic acid and 2-hydroxybenzoic acid concentrations in *E. coli*. The system was applied to screen active enzymes capable of converting benzaldehyde and 2-hydroxybenzoaldehyde to their carboxylic acid derivatives (van Sint Fiet et al., 2006). Since then, several successful applications of biosensors have been described in *E. coli* (Selvamani et al., 2017; Ganesh et al., 2019) and more recently in cell-free systems (Eggeling et al., 2015; Voyvodic et al., 2019). However, transferring genetic sensors between various organisms remains highly challenging. For example, the simple transfer of a tetracycline resistance gene circuit from yeast to mammalian cells required extensive optimizations such as the translation of the reporter, the DNA sequences of the heterologous proteins, the nuclear localization signal of the transcription factor and the design of the promoter (Nevozhay et al., 2013).

In yeast, one of the first designed sensing systems was used to monitor the intracellular S-adenosylmethionine concentrations (Umeyama et al., 2013). Transcriptional-based biosensors were also constructed to screen for muconic acid-producing yeast strains (Leavitt et al., 2017; Snoek et al., 2018). Other types are now implemented, using optogenetic regulation such as the blue light-activated EL222 from *Erythrobacter litoralis* that was recently reported to control, in yeast, the mitochondrial isobutanol-producing pathway (Zhao et al., 2018).

4-hydroxybenzoic acid (pHBA), a molecule produced from chorismate by chorismate lyase, is present in low amounts in bacterial cultures (Winter et al., 2014). pHBA is essential in all organisms for coenzyme Q synthesis (Tran and Clarke, 2007) but was found to inhibit yeast growth when added to the culture medium (Ando et al., 1986; Palmqvist et al., 1999; Larsson et al., 2000). pHBA-derived natural products form a large group of secondary metabolites that exhibit a wide variety of biological activities (Wang et al., 2018). Industrial uses of pHBA include manufacturing of liquid crystal polymers and thermoplastics used for space technologies (Rothschild, 2016). The alkyl esters of pHBA, namely parabens, are also widely used as preservatives in drugs, cosmetic products and food products. However, their toxicity is a major human health concern (Giulivo et al., 2016). Currently, pHBA is chemically synthesized from petroleum-derived building blocks. Biotechnology offers an alternative for pHBA production, based on the shikimate pathway (Lee and Wendisch, 2017). Recently, researchers have engineered yeast to overproduce pHBA, increasing the flux to chorismate and expressing, in the modified strain, the chorismate lyase gene (UbiC) from *E. coli* (Averesch et al., 2017).

Therefore, pHBA is a molecule for which *in vivo* biological detection is critical, and a performant pHBA biosensor could be used either to build detection kits capable of operating in various biological environments or to screen the capacity of modified strains to overproduce pHBA. In this study, we report the construction of a synthetic transcription factor (sTF) capable of sensing pHBA, *in vivo*, in *Saccharomyces cerevisiae*. We tested the transcriptional activity against the native effector of *R. palustris* HbaR and a battery of metabolites with similar structure and determine the promiscuity and binding characteristics of each of them. Last, we exemplified the activity of our newly constructed biosensor in a pHBA-producing strain.

## Materials and Methods

### Culture Conditions

The vectors described in this work were constructed in the *E. coli* DH5α strain using standard molecular biology protocols (Sambrook and Russell, 2001). The CEN.PK 2C-1 *S. cerevisiae* strain (*MATa*; *ura3-52*; *trp1-289*; *leu2-3, 112*; *his3:1*; *MAL2-8C*; *SUC2*) was used throughout the entire study.

For the screening experiments, yeast strains were grown in synthetic medium containing 20 g/L of glucose, 6.9 g/L of yeast nitrogen base with or without pABA and Complete Supplement Mixture (CSM) drop-out minus uracil and histidine (Formedium). The different inducers were dissolved in the medium and pH was then adjusted to 5.5. All culture media were sterilized by filtration. For pHBA production, the synthetic medium without pABA and with CSM drop-out minus His, Ura, and Leu was supplemented with 76 mg/L tyrosine, phenylalanine and tryptophan.

Yeast strains were cultured as follows. A fresh yeast colony from a SD minus Ura and His agar plate containing pABA was resuspended in water to an OD_600nm_ of 0.2 and 5 μL of this solution was used to inoculate 200 μL of media. Cells were grown at 30°C in a MixMate (Eppendorf) with 900 rpm agitation for 20 h. For the pHBA production experiments, a fresh colony was resuspended in water to an OD_600nm_ of 0.2 and 50 mL of culture medium was inoculated with 400 μL of this solution. Cells were grown at 30°C with an agitation of 200 rpm in an INFORS incubator (25 mm orbital) for 65 h.

### Vectors and Strains Constructions

All plasmids and strains used in this study are listed in Supplementary Tables 1, 2. Yeast transformation was performed using the high efficiency protocol from Gietz (2014). The *hbaR* gene from *Rhodopseudomonas palustris*, the native *ubiC* gene from *E. coli* and the modified *ARO4K226L* gene from *S. cerevisiae* were codon-optimized for *S. cerevisiae* (Twist Bioscience). All DNA sequences can be found in Supplementary Table 3.

The pHBA sTF was constructed using the vector FRP880 as a backbone. FRP880 was digested with *Eco*RV to remove the estradiol-sensing domain and the HbaR-sensing domain was inserted by blunt-end cloning leading to the plasmid pSCC185. To create the ySCC185-F strain, we first integrated a DNA fragment expressing mKATE2 under the control of the *TDH3* promoter into the locus 2 of chromosome X (Mikkelsen et al., 2012) and carrying the auxotrophic marker *TRP1*, yielding ySCC001. pSCC185 was then linearized with the restriction enzyme *Pac*I, integrated in the *HIS3* locus in ySCC001, yielding ySCC185. Finally, the linearized plasmid FRP795, containing a promoter with 8 LexA DNA binding domains and a *CYC1* minimal promoter controlling the expression of the mCitrine fluorescent protein was integrated in ySCC185, yielding ySCC185-F. Integrations were verified by colony PCR and by functional analysis.

To build the pHBA-overproducing strain, we used the vector pENZ030 containing two homologous arms for the integration in the *HO* locus, a *LEU2* auxotrophic marker and a bidirectional promoter formed by *TDH3* and *PGK* and flanked by *ADH1* and *ADH2* terminators. The *ubiC* ORF was cloned downstream of the *TDH3* promoter by cutting pENZ030 with *Xho*I. The *ubiC* gene was inserted in pENZ030 by isothermal assembly. The pHBA-producing strains were derived from ySCC185-F. First, the *TRP3* gene was deleted using a hygromycin resistance cassette from the vector pUG75 flanked by a 38 bp homologous region of *TRP3* yielding ySCC185-F-T. The *ARO7* gene was then deleted and its promoter used to express the *ARO4K229L* mutant (Williams et al., 2015). To construct this, the previous strain was transformed with two cassettes. The first one contained the gene encoding the *ARO4K229L* mutant with two homologous regions of 40 bp, upstream for *ARO7* and downstream for the second integration cassette. The second cassette contained the *CYC1* terminator and G418 resistance. This DNA was amplified from the vector pMRI34 and the PCR containing homologous regions for the first cassette and the end of *ARO7* gene yielding ySCC185-F-A. This last strain was used to express the *E. coli ubiC* gene, using a PCR amplified DNA fragment and the vector pENZ030-UbiC as a template, yielding ySCC185-UbiC. The control strain for the pHBA production experiments was derived from ySCC185-F-A with an integrated copy of the empty vector pENZ30 (ySCC185-30).

### Flow Cytometry

Cells were incubated for 30 min with 70 μg/mL cycloheximide (prepared in DMSO), further diluted to reach a cell count between 0.5 × 10^6^ and 1.5 × 10^6^ cells/mL and then immediately injected into a flow cytometer MACSQuant VYB (Miltenyi Biotec, Germany). Regions were determined as a function of the mCitrine and mKate2 fluorescence. Optimal laser and filter setups for the two dyes were as follows: 488 nm laser and 525/25 Band Pass B1-filter for mCitrine, and 561 nm laser and 615/10 Band Pass Y2-filter for mKate2. The expression profile of mKate2 was measured for each sample by the MACSQuant VYB flow cytometer with the MACSQuantify TM Software (Miltenyi Biotec, Germany). A filter was applied on FSC-A/SSC-A to select homogeneous cells regarding size, shape, and cellular complexity. The mean fluorescence value of mKate2 and mCitrine was calculated and exported.

### Western Blot

Protein extracts were prepared following the protocol described by Zhang et al. (2011). Briefly, 1 OD_600nm_ of pelleted cells were pre-treated with 100 μL of 2 M lithium acetate solution, left standing on ice for 5 min, followed by 5 min centrifugation at 5,000 g, 4°C. The supernatant was discarded and 100 μL of a 0.4 M solution of NaOH added. After gentle resuspension and 5 min standing on ice, samples were centrifuged 5 min at 4°C. Cell pellets were vigorously vortexed with 60 μL of a dye solution containing bromophenol-blue and supplemented with 5% β-mercaptoethanol. After denaturation at 99°C for 10 min, 10 μL of each sample was deposited on a 10% SDS page gel. Semi-dry transfer was performed on a PVDF membrane (Merck Millipore, Darmstadt, Germany) using a Trans-Blot® SD Cell BioRad apparatus (15 V at 600 mA during 30 min). Five percent powdered milk in TBS was used as a blocking agent. Mouse primary antibody anti-EGFP (Thermoscientific), and secondary anti-mouse IgG coupled with alkaline phosphatase (Thermoscientific) were diluted following instructions from the provider. Antibody incubations were performed in 5% powdered milk in TBS during 1 h. Proteins were detected by the incubation of BCIP/NBT AP substrate buffer (Sigma-Aldrich, St. Louis, MO, USA).

### pHBA HPLC Analysis

Supernatants of pHBA-producing strains were collected during 3 days of batch growth. Analysis of the pHBA production was carried out on a Waters Alliance HPLC system coupled with a 996 Waters PDA detector. Twenty microliter of the culture supernatant was injected on a Xterra column (100 × 2.0 mm and 3 μm particle size) maintained at 45°C during the analysis. The mobile phases consisted in a mixture of A (H_2_O with 0.1% formic acid) and B (acetonitrile with 0.1% formic acid). The flow was set at 1 mL/min with the following gradient: 0–0.1 min 100% A, 0.1–11 min 40% A and 60% B, 11–12.5 min 100% B, 12.5–13 min 100% A. Absorbance spectra from 210 to 400 nm were recorded. pHBA was quantified by its absorbance at 254 nm.

### Statistical Analysis

Statistical analyses were performed using the GRAPHPAD PRISM 8.2 software.

## Results and Discussion

### Design and Validation of the Synthetic Transcription-Based Biosensor

**Figure 1:**
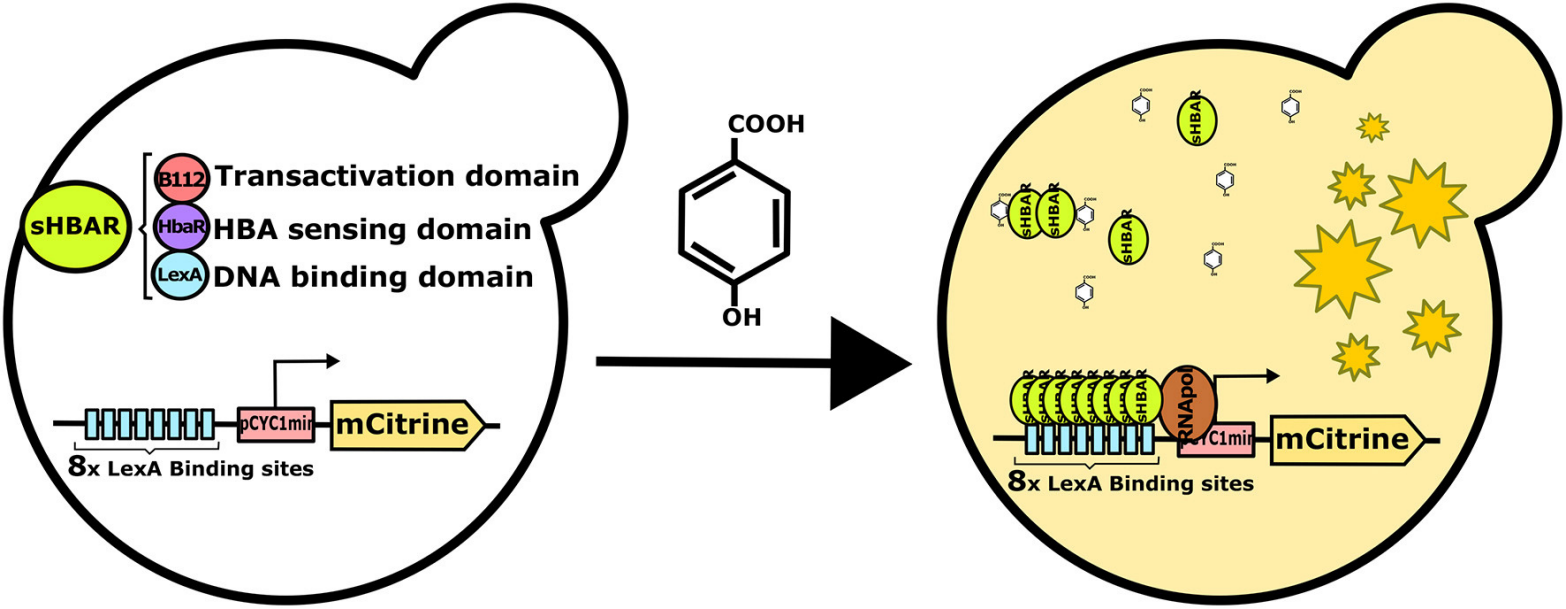
Schematic representation the sHbaR sTF detecting pHBA *in vivo* in *S. cerevisiae*.

The architecture chosen for the pHBA sTF was inspired by the work performed by Stelling and co-workers, in which a collection of modular tripartite sTFs, activated by estradiol, were developed (Ottoz et al., 2014). We thus designed our sTF using LexA and B112 proteins as DNA binding and transactivation domains, respectively (Figure 1). We cloned the ORF of the HbaR ligand binding domain from *R. palustris*, known to be specifically activated by pHBA (Egland and Harwood, 2000), between the transactivation and DNA binding domains (pSCC185, see Supplementary Table 2). To report the transcriptional activation of our synthetic pHBA biosensor, designated as sHbaR, we used the previously constructed vector FRP795 containing the gene encoding for the mCitrine yellow fluorescent protein downstream of the minimal *CYC1* promoter with 8 LexA DNA binding sites (Ottoz et al., 2014). The resulting strain, ySCC185-F, bears both the reporter gene and the sHbaR sTF.

**Figure 2:**
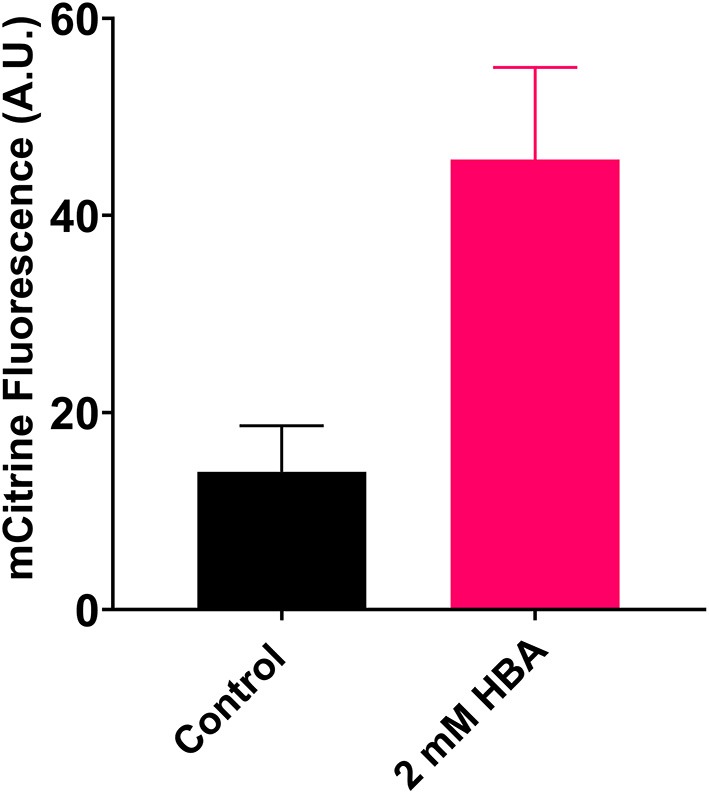
pHBA activation of sHbaR in *S. cerevisiae*. mCitrine fluorescence was measured by flow cytometry at late exponential phase. Values are calculated as the mean of three biological replicates.

To validate transcriptional activation by pHBA, the strain ySCC185-F was cultured in 96-well plates at 30°C with or without pHBA (2 mM). mCitrine fluorescence was measured at late exponential phase (OD_600nm_ = 4) to get the best sensitivity possible with a rather small volume of culture. As shown in Figure 2, the mCitrine fluorescence is 3-fold higher with pHBA compared with the control medium that did not contain the inducer. We also verified that the measured fluorescent signal is proportional to the quantity of mCitrine measured by Western Blot (Supplementary Figure 1). Notwithstanding the fact that a previous *in vitro* study described a sTF activated by pHBA (Yao et al., 2018), to our knowledge, sHbaR is the first biosensor that can be activated by pHBA *in vivo*, in *S. cerevisiae*.

### pABA Also Activates the sHbaR Sensing System

Analysis of the first experiments demonstrated that the fluorescent signal measured with the strain ySCC185-F in the absence of pHBA is greater than the signal measured without the reporter (Supplementary Figure 2A). This could be due either to some leakage of the synthetic promoter in the absence of the sTF or promiscuous activation of sHbaR by molecule(s) present in the medium or in the cells. In fact, promiscuity of benzoic acid-responsive natural transcription factors has been reported before, evidencing that they can respond, to different degrees, to a variety of substituted benzoic acids (but not pHBA) (Xue et al., 2014). Therefore, we hypothesized that a compound present in the synthetic medium could be also an effector of sHbaR.

**Figure 3:**
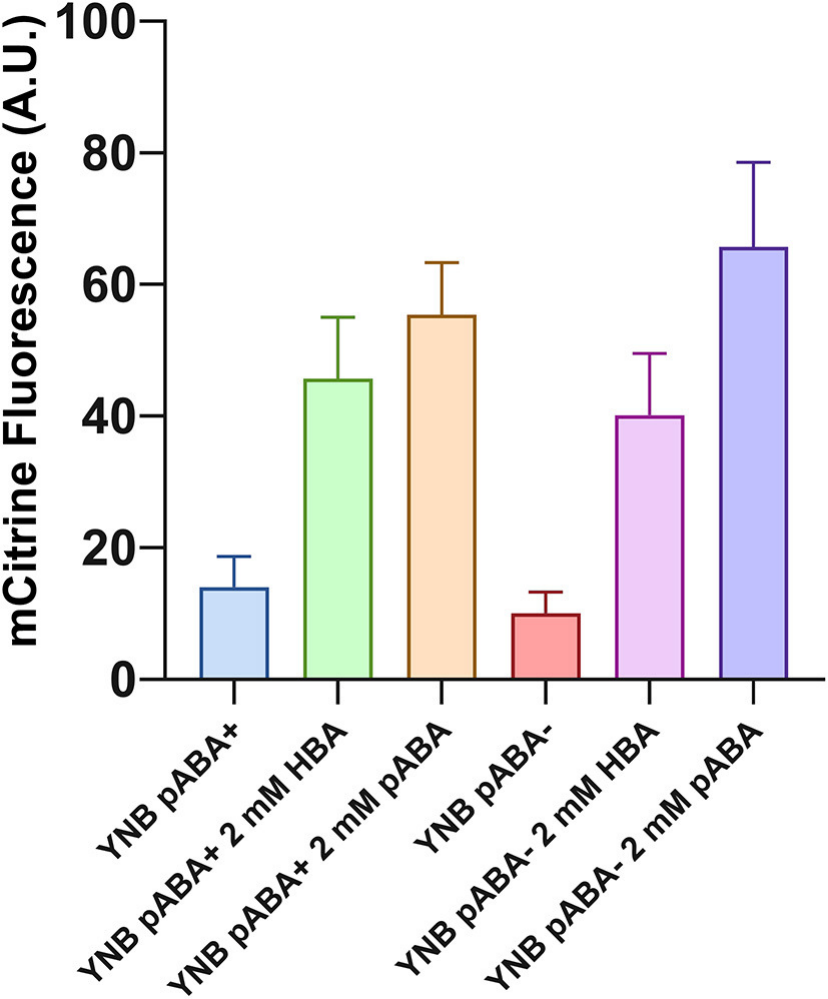
pABA activates sHbaR. ySCC185-F was grown with YNB containing or not pABA and with the addition, in the medium, of 2 mM of pHBA or pABA. Cells were harvested in late exponential phase. Values are calculated as the mean of three biological replicates.

The most similar metabolite to pHBA found in our synthetic media is p-aminobenzoic acid (pABA), which, according to the supplier's medium composition, is present at a final concentration of 1.46 μM. We thus analyzed if the low levels of pABA in this medium could activate sHbaR. As expected, the fluorescence signal produced by the strain ySCC185-F is reduced by almost 30% in a synthetic medium devoid of pABA compared to the same one containing pABA (*p*-value 0.0014, Figure 3). This indicates that a rather small concentration of this metabolite in the growth medium indeed activates sHbaR *in vivo*. We also tested if tyrosine and phenylalanine that possess rather similar chemical structures to pHBA and that are present in the CSM at a concentration of 300 μM each could also activate sHbaR. We grew ySCC185-F with and without these amino acids and observed no difference between both conditions (Supplementary Figure 2B). We also supplemented each of the two amino acids to a final concentration of 2 mM and could not observe any difference in the level of fluorescence compared with the control medium (Supplementary Figure 2B). Since the promoter-reporter construction that we have used showed absolutely no leakage of transcription in the absence of inducers in a previous study (Ottoz et al., 2014), we thus attributed the remaining fluorescence signal to the presence of other molecules capable of activating sHbaR.

To further understand the possible competition or synergy between pABA and pHBA, we grew the strain ySCC185-F in YNB (containing or not pABA) in the presence of externally added pABA or pHBA (2 mM) (Figure 3). When pABA is present in the medium, the addition of 2 mM of pHBA or pABA induced, respectively, a 3- or 4-fold fluorescence increase. The same trend is observed on a synthetic medium devoid of endogenous pABA, i.e., a 4-fold augmentation of fluorescence with 2 mM pHBA and 6-fold with 2 mM pABA. We thus hypothesize that there is no competition or synergy between these two metabolites and sHbaR and that pABA is simply a better effector for sHbaR than pHBA.

### Promiscuity of the Synthetic sHbaR Sensing System

**Figure 4:**
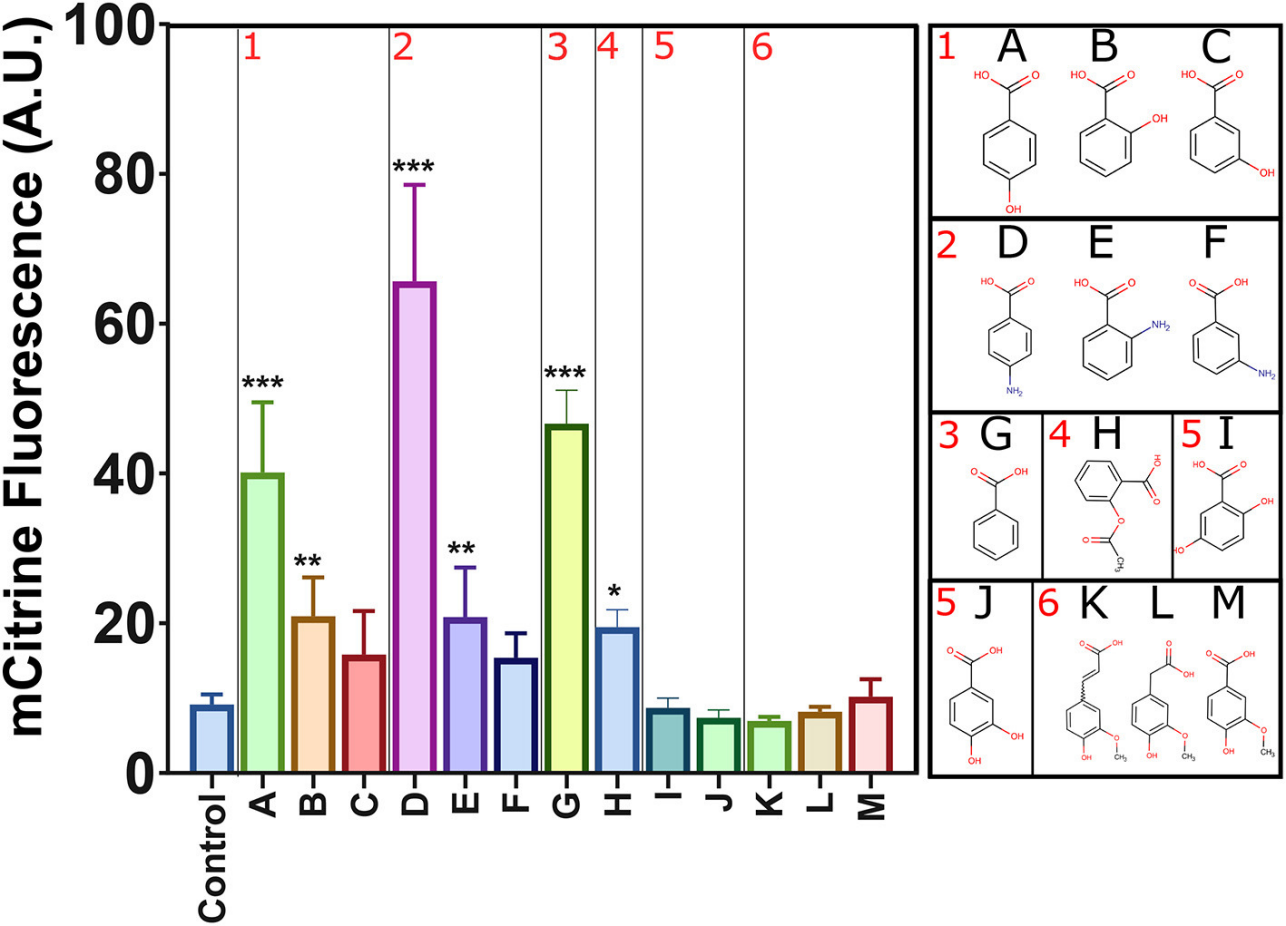
sBAD activity with different benzoic acid derivatives. mCitrine fluorescence was measured by flow cytometry. Values are calculated as the mean of three biological replicates. The different metabolites are grouped by similarity. (1) mono hydroxybenzoic acids (A pHBA, B 2HBA, C 3HBA), (2) mono aminobenzoic acids (D pABA, E 2NBA, F 3NBA), (3) (G) benzoic acid, (4) (H) aspirin, (5) dihydroxybenzoic acids (I 2,5DHBA, J 3,4DHBA), and (6) longer radical in position 1 of the benzoic ring (K Ferulic acid, L Homovanillic acid, M Vanillic acid). Represented values are the average of the mean fluorescence measured (*n* = 10). Error bars indicate the standard deviation of the measurements (*n* = 10). Statistical tests (Dunnett's multiple comparisons test) were performed to calculate differential significance between inducers and the control condition (***p < 0.0001 or **p < 0.001 or *p < 0.01).

Since sHbaR seems to not completely distinguish between two different substituents at the *para* position of benzoic acid, we decided to change the name of our biosensor to sBAD (previously known as sHbaR), standing for “sensor of benzoic acid derivatives.” We further investigated the promiscuity of sBAD with different benzoic acid-derived chemicals at 2 mM each, in the absence of pABA in the growth medium (Figure 4). Interestingly, benzoic acid itself leads to a 5-fold activation of sBAD, a value comparable to the one obtained with pHBA (~4 fold) and pABA (~6 fold). The other three molecules substituted in the *ortho* position (2HBA (salicylic acid), 2NBA (anthranilic acid), and aspirin) were able to produce a low, but significant (2-fold) response of sBAD. A general trend for the specificity is remarkably conserved between hydroxy- or amino-substituted benzoic acid derivatives: the *para* position is the preferred one, followed by the *ortho* position and the *meta* position, which is the least potent for sBAD activation. For the rest of the tested metabolites, only those mono-substituted with a hydroxyl or amino group at the *ortho* or *para* position of the benzoic ring provided a significant increase of fluorescence compared to the control. Surprisingly, a larger substituent at the *ortho* position (aspirin, Figure 4H) can also activate sBAD. On the contrary, all other di-substituted benzoic acid derivatives were incapable of activating sBAD. This result indicates that sBAD has a rather tight control of the number of substituents on the aromatic ring.

It is known that the positions and the chemical properties of benzoic acid substituents influence the pK_a_ of the carboxylic group of the corresponding chemicals (Supplementary Table 4). A difference of 1.5 pH units indeed exists between the *ortho*-hydroxybenzoic acid (pK_a_ = 3) and pHBA (pK_a_ = 4.6). Thus, all tested metabolites have different pK_a_s and their diffusion through the plasma membrane of yeast might be slightly different. Moreover, it is known that the rates of import of benzoic acid derivatives (*p*-coumaric acid and pHBA) in *S. cerevisiae* are different (Barnhart-Dailey et al., 2019). However, as pHBA, 2HBA, pABA, 2NBA, benzoic acid, and aspirin all activate sBAD *in vivo* under the conditions tested, we assume that they are transported in the cell in order to activate the intracellular sBAD and trigger the corresponding fluorescence signal. Alternatively, the diverse responses obtained could also originate from different binding affinities of the different compounds to the sTF. As no crystal structure of the native transcription factor HbaR from *R. palustris* with its cognate binder (pHBA) is available, we can only hypothesize that the delocalization of π-electrons of the aromatic benzene ring may partly control the recognition of the inducer by the binding domain of the sBAD biosensor.

### Dynamic Properties of the sBAD Sensing System

**Figure 5:**
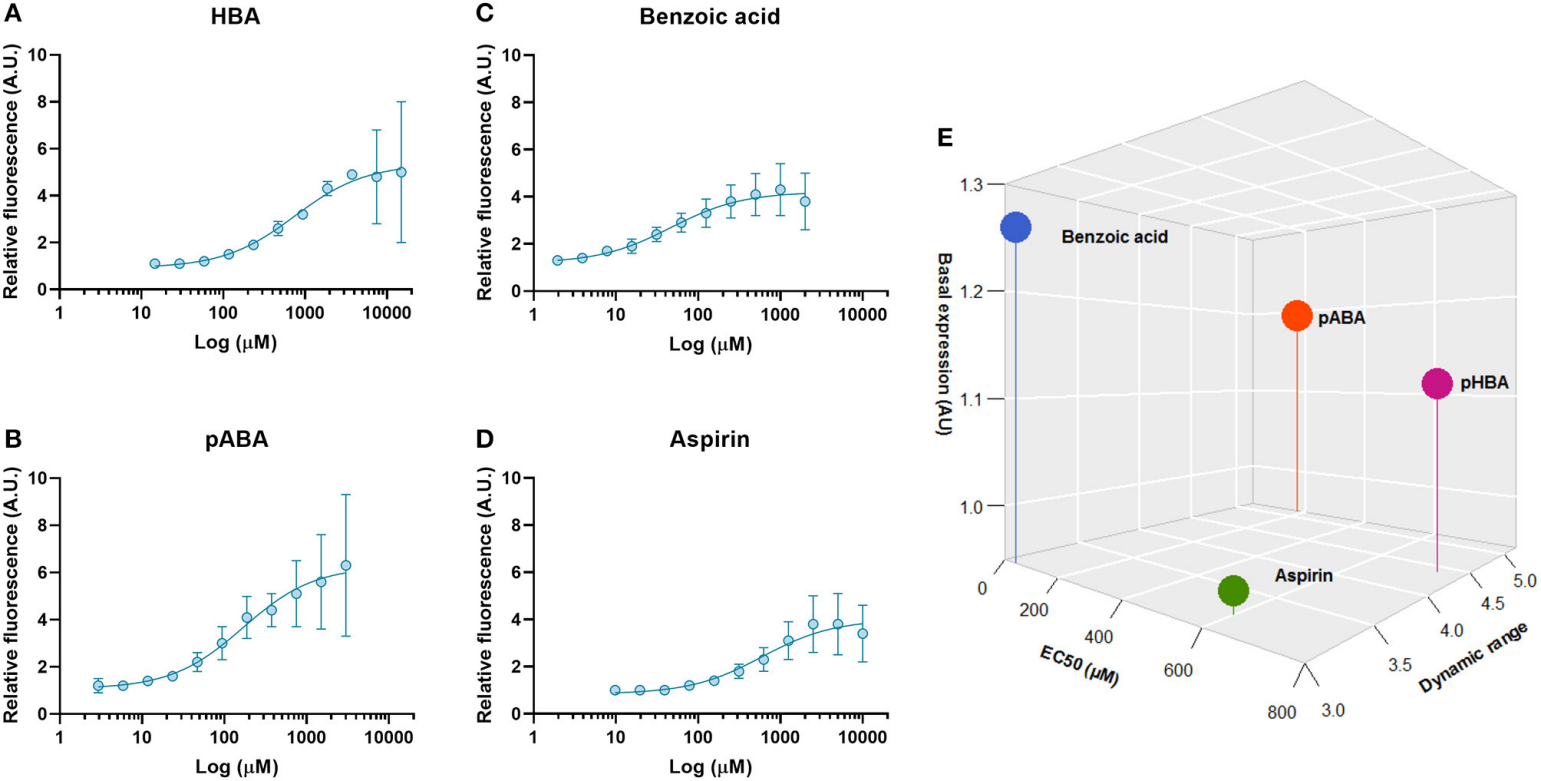
Dose-response curves obtained with the strongest benzoic acid derivative effectors of sBAD expressed in *S. cerevisiae*. mCitrine fluorescence was measured by flow cytometry. The relative fluorescence of mCitrine was measured with cells grown at different concentration of **(A)** pHBA, **(B)** pABA, **(C)** Benzoic acid, and **(D)** Aspirin is compared with the control condition. In **(E)**, the affinity and dynamic parameters calculated from different the curves dose response are presented. Values are calculated as the mean of three biological replicates.

To obtain a more accurate resolution of the cellular responses and the operational dynamic range of the most active inducers, we quantified the binding affinity using dose-response curves with the yeast-expressed sBAD (Figures 5A–D). For the metabolites 2HBA and 2NBA, we could not determine the different parameters since the signal never reached saturation at the highest concentrations tested (Supplementary Figure 3). This was probably due to some growth defect of our strain cultured with 2HBA at a concentration over 2 mM and the difficulty in dissolving 2NBA at concentrations higher than 3 mM.

In our experimental conditions, pABA and benzoic acid induced sBAD at much lower concentrations than pHBA which is supposed to be the natural inducer of HbaR in *R. palustris*. The EC50 for pHBA is 4- and 15-fold higher than the ones measured for pABA and benzoic acid, respectively (Table 1, Figure 5E). It should be noted that both the EC50 and dynamic range values were slightly smaller with aspirin than with pHBA. The biggest dynamic range was found with pABA, which in any case showed the best performance as an effector compared to the three others tested activators (Figure 5E). Two hypotheses can explain our results. The first one relates to the three-dimensional structure of sBAD. In our design, the effector binding domain was inserted between two other proteins, thus possibly changing the global fold of the HbaR sensing domain and consequently, the specificity or affinity of sBAD for alternate molecules such as pABA. The second hypothesis is that the native bacterial HbaR transcription factor could be promiscuous. For example, ligand promiscuity has been observed with the transcription factor XylR of *Pseudomonas putida* which regulates genes involved in the metabolism of aromatic compounds and can be activated by toluene, *m*-xylene, or benzene (Galvão and de Lorenzo, 2006). Even more interesting, the transcription factor BenM from *Acinetobacter baylyi* bears not one but two binding pockets in its structure, one for *cis,cis*-muconic acid and the second for benzoic acid (Craven et al., 2009). HbaR from *R. palustris* belongs to the CRP-FNR transcription factor family that do not display ligand promiscuity compared with the XylR-NtrC or LysR families (Egland and Harwood, 2000). It should be noted, however, that CRP from *P. putida* can be activated by cyclic AMP as well as cyclic GMP, albeit with a lower affinity (Arce-Rodríguez et al., 2012). All this information suggests that pHBA may not be the only physiological binder of HbaR in *R. palustris*.

**Table 1:** Affinity and dynamic range parameters of sBAD with the inducers HBA, pABA, benzoic acid, and aspirin.

**Metabolite**	**EC50 (μM)**	**Dynamic range**
HBA	749 ± 399	4.4 ± 0.5
pABA	169 ± 81	5.2 ± 0.6
Benzoic acid	47 ± 20	3.0 ± 0.3
Aspirin	598 ± 283	3.2 ± 0.3

*Reported values are calculated as the mean of three biological replicates ± SD*.

### *In vivo* Application of sBAD in pHBA-Overproducing Yeast

**Figure 6:**
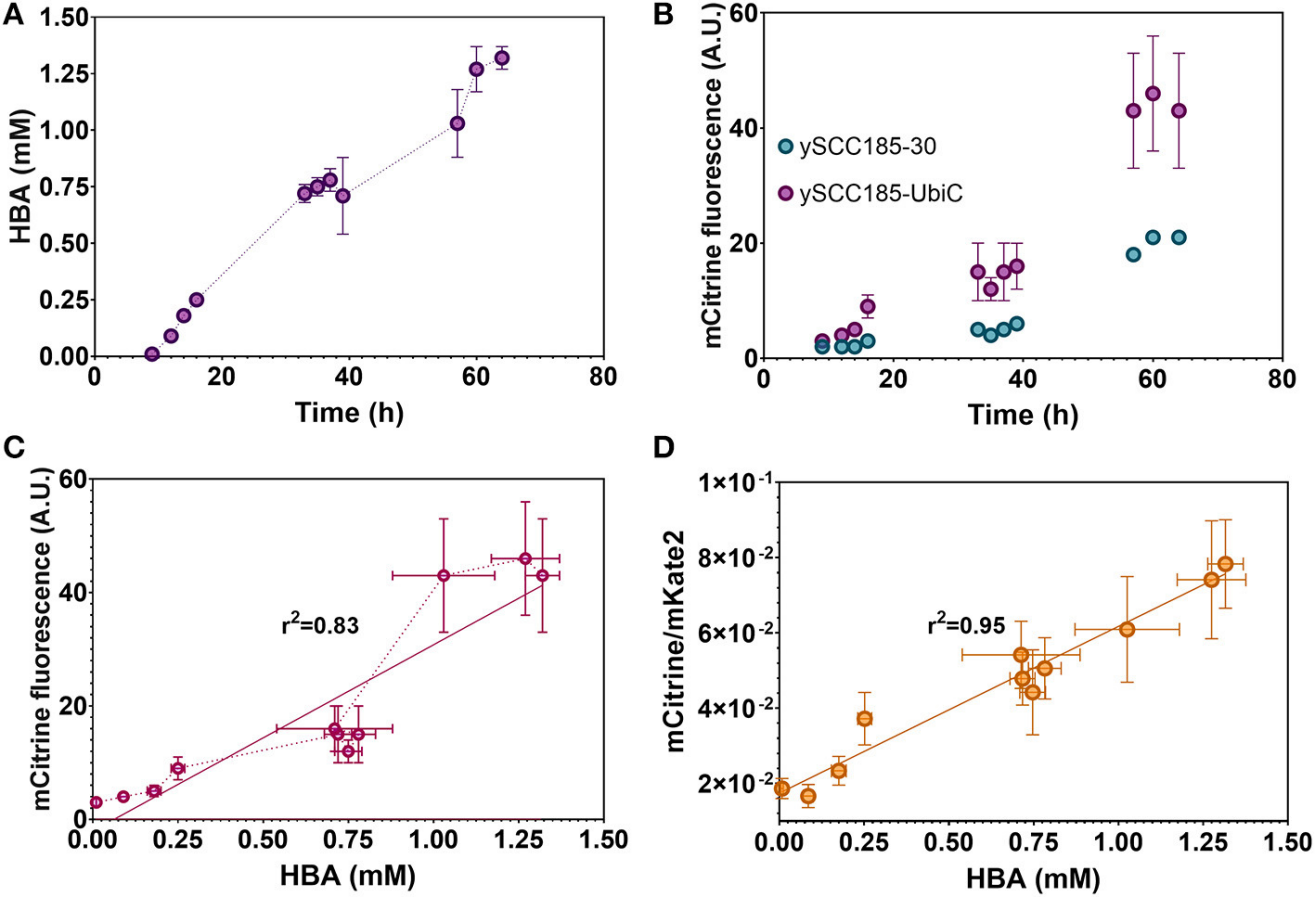
sBAD functions in an engineered *S. cerevisiae* strain producing pHBA. **(A)** pHBA production during 65 h of growth in a medium devoid of pABA. **(B)** mCitrine fluorescence during culture in strains ySCC185-30 and ySCC185-UbiC. **(C)** Correlation between the mCitrine fluorescence and pHBA production in ySCC185-F UbiC. **(D)** Correlation between the ratio of mCitrine over mKATE2 fluorescence with pHBA production. Values are calculated as the mean of three biological replicates.

We next addressed whether sBAD would support real-time monitoring of *in vivo* pHBA metabolite production. To do so, we engineered the ySCC185-F strain to overproduce pHBA as previously described (Averesch et al., 2017). We first deleted the *TRP3* gene and then substituted the wild-type version of *ARO7* with the *ARO4K229L* gene. This strain named ySCC185-F-A can produce higher yields of chorismate compared to the ySCC185-F strain (Averesch et al., 2017). ySCC185-F-A was then further modified to express the *ubiC* (chorismate lyase) gene from *E. coli* under the constitutive promoter *TDH3*, leading to the strain ySCC185-UbiC. We then assessed the number of cells, fluorescence of mCitrine and mKate2 (reporter of the cell number) for 65 h of cultivation with the newly constructed strains ySCC185-UbiC and ySCC185-30 (control strain). Both strains showed similar specific growth rates, indicating that the achieved level of pHBA production does not alter cell physiology (Supplementary Table 5). After 65 h of cultivation, the strain ySCC185-UbiC produced 1.32 mM of pHBA while the control strain ySCC185-30 did not produce any detectable amounts of it (Figure 6A). The production yield achieved in our strain is half of that reported by Averesch et al. (2017). This difference might be attributed to the modifications included in our parental strain (ySCC185-F) or differences in the media composition/culture conditions. Nevertheless, the concentrations reached during the cultures are in the range of detection of our biosensor, making it useful for the proof of concept of its potential use in metabolic engineering.

We then analyzed the response of sBAD to the internal production of pHBA in a medium devoid of pABA. As previously seen with ySCC185-F, a residual fluorescence signal is observed in the control strain ySCC185-30 and this signal increases with time (Figure 6B). However, mCitrine fluorescence is always higher with ySCC185-UbiC than with ySCC185-30. The accumulation of mCitrine after 60 h in ySCC185-30 and ySCC185-UbiC is probably due to a continuous production of the fluorescent protein, even after cell growth arrest. In fact, even the pHBA production increases after 60 h of culture while the cell count does not change anymore after 40 h (Supplementary Figure 4). Remarkably, there is a good linear correlation (*R*^2^ = 0.83) between the mCitrine fluorescence signal and pHBA amounts produced by ySCC185-F UbiC cells (Figure 6C). More importantly, if we plot the ratio of the mCitrine fluorescence values (representative of sBAD activity) and the mKate2 fluorescence values (representative of the cell count) against pHBA concentrations obtained in the production experiments, the linearity of the response is even greater (*R*^2^ = 0.95, Figure 6D, Supplementary Figure 5).

Therefore, our synthetic biosensor sBAD is functional and accurate enough to detect and measure the *in vivo* production of pHBA and, as such, can be used to screen for strains producing high titers of pHBA (up to 10 mM) in batch cultures. Furthermore, the capacity of sBAD may not be restricted to screening pHBA overproduction in yeast but can also be applied to pABA, benzoic acid and, to a lesser extent, other *ortho*-monosubstituted benzoic acid derivatives.

## Conclusions

This work has presented the successful design, construction and characterization of an orthogonal benzoic acid derivative biosensor (sBAD) in *S. cerevisiae* using the binding domain of the bacterial HbaR from *R. palustris* linked to the LexA protein as a DNA binding domain and B112 as a transactivation domain. This biosensor system allowed us to easily monitor, in real-time, a fluorescence signal linearly correlated to extracellular concentrations of pHBA. The promiscuity of the biosensor in yeast was also analyzed and, quite unexpectedly, pABA and benzoic acid also produce responses that are even more pronounced than the one measured with pHBA, notably in terms of sensitivity and dynamic range (for pABA). The relatively strong effect of the substituent position (*para* > *ortho* > *meta*), rather than the chemical properties of the substituent itself, indicate that the physicochemical properties that govern the promiscuous recognition of benzoic acid derivatives by sBAD are non-trivial. As our output signal is easily measurable, and considering the strong linearity obtained between our reporter signals and pHBA concentrations, our biosensor opens the way to a more thorough study of its binding properties, for instance with random mutagenesis approaches. This novel biosensor for detecting benzoic acid derivatives can be considered a useful tool to improve the production of production of such derivatives by screening large populations of yeast mutants. Moreover, the strong recognition of pABA or benzoic acid by sBAD could also be used for medical sensing purposes in bodily or chemical fluids, allowing an easily measurable output signal (fluorescence) to be monitored over a range of fluid concentrations covering three orders of magnitude.

## Data Availability Statement

The datasets generated for this study are available on request to the corresponding author.

## Author Contributions

J-LF and GT conceived this project. SC-C designed the experiments, carried out experimental work, analyzed, and interpreted the data. MF contributed to the analysis of the data. SC-C, GT, and PU wrote the paper. All authors assisted in this process.

### Conflict of Interest

The authors declare that this study received funding from Toulouse White Biotechnology. The funder was not involved in the study design, collection, analysis, interpretation of data, the writing of this article or the decision to submit it for publication. The authors declare that the research was conducted in the absence of any commercial or financial relationships that could be construed as a potential conflict of interest.

## Abbreviations

bp: base pair
pHBA: 4-hydroxybenzoic acid
pABA: 4-aminobenzoic acid
2HBA: 2-hydroxybenzoic acid (salicylic acid)
3HBA: 3-hydroxybenzoic acid
2NBA: 2-aminobenzoic acid (anthranilic acid)
3NBA: 3-aminobenzoic acid
2,5DHBA: 2,5 dihydroxybenzoic acid (gentisic acid)
3,4DHBA: 3,4 dihydroxybenzoic or protocatechuic acid
CSM: Complete Supplement Mixture
SD: Synthetic Defined medium
sTF: Synthetic transcription factor
sBAD: sensor of benzoic acid derivatives
YNB: Yeast Nitrogen Base.
