# Supplementary material for: Development of a Biosensor for Detection of Benzoic Acid Derivatives in Saccharomyces cerevisiae
Source: Front Bioeng Biotechnol. 2020 Jan 7;7:372. doi: 10.3389/fbioe.2019.00372 (PMC6959289; doi:10.3389/fbioe.2019.00372)
Supplement: Supplementary file 1 [file Data_Sheet_1.pdf]

SUPPLEMENTARY MATERIAL

SUPPLEMENTARY TABLES

Supplementary Table 1. Genotypes of the various *S. cerevisiae* strains used in this study.

Strain name	Genotype	Parental strain	Source
S. cerevisiae CEN.PK2-1C	*MATa; his3D1; leu2-3_112; ura3-52; trp1-289; MAL2-8c; SUC2*	-	Euroscarf
ySCC001	*X-II:: TDH3p-mKate2-TRP1*	*S. cerevisiae* CEN.PK2-1C	This study
ySCC185	*X-II:: TDH3p-mKate2-TRP1*<br>*HIS3:: $_{P}$ACT1(1-520)-LexA-sHBA-haB112-$_{T}$CYC$_{1}$*	ySCC001	This study
ySCC185-F	*X-II:: TDH3p-mKate2-TRP1*<br>*HIS3:: $_{P}$ACT1(1-520)-LexA-sHBA-haB112-$_{T}$CYC$_{1}$*<br>*URA3::(lexA-box)$_{8}$-$_{P}$minCYC$_{1}$-mCitrine-$_{T}$CYC$_{1}$*	ySCC185	This study
ySCC185-F-T	*X-II:: TDH3p-mKate2-TRP1*<br>*HIS3:: $_{P}$ACT1(1-520)-LexA-sHBA-haB112-$_{T}$CYC$_{1}$*<br>*URA3::(lexA-box)$_{8}$-$_{P}$minCYC$_{1}$-mCitrine-$_{T}$CYC$_{1}$*<br>*Δtrp3:: hphMX*	ySCC185-F	This study
ySCC185-F-A	*X-II:: TDH3p-mKate2-TRP1*<br>*HIS3:: $_{P}$ACT1(1-520)-LexA-sHBA-haB112-$_{T}$CYC$_{1}$*<br>*URA3::(lexA-box)$_{8}$-$_{P}$minCYC$_{1}$-mCitrine-$_{T}$CYC$_{1}$*<br>*Δtrp3:: hphMX*<br>*Δaro7::ARO4K229L- tCYC1-kanMX*	ySCC185-F-T	This study
ySCC185-30	*X-II:: TDH3p-mKate2-TRP1*<br>*HIS3:: $_{P}$ACT1(1-520)-LexA-sHBA-haB112-$_{T}$CYC$_{1}$*<br>*URA3::(lexA-box)$_{8}$-$_{P}$minCYC$_{1}$-mCitrine-$_{T}$CYC$_{1}$*<br>*Δtrp3:: hphMX*<br>*Δaro7::ARO4K229L- tCYC1-kanMX*<br>*HO::TDH3p-ADH1t-LEU2*	ySCC185-F-A	This study
ySCC185-UbiC	*X-II:: TDH3p-mKate2-TRP1*<br>*HIS3:: $_{P}$ACT1(1-520)-LexA-sHBA-haB112-$_{T}$CYC$_{1}$*<br>*URA3::(lexA-box)$_{8}$-$_{P}$minCYC$_{1}$-mCitrine-$_{T}$CYC$_{1}$*<br>*Δtrp3:: hphMX*<br>*Δaro7::ARO4K229L- tCYC1-kanMX*<br>*HO::TDH3p-ADH1t-LEU2*<br>*HO::TDH3p-EcUbiC-ADH1t-LEU2*	ySCC185-F-A	This study

Supplementary Table 2. Plasmids used in this study.

Strain name	INSERT	Marker	Integration site	Source
FRP795	*(lexA-box)$_{8}$-$_{P}$minCYC$_{1}$-Citrine-$_{T}$CYC$_{1}$*	URA3	URA3	Addgene
FRP880	*$_{P}$ACT1(1-520)-LexA-ER-haB112-$_{T}$CYC$_{1}$*	HIS3	HIS3	Addgene
pHES854	*TDH3p-mKATE2*	HIS3	-	Addgene
pMRI34	*Geneticin resistance*	Geneticin	-	(Xie et al., 2014)
pUG75	*Hygromycin resistance*	hphMX	-	Euroscarf
pSCC185	*$_{P}$ACT1(1-520)-LexA-sHBA-haB112-$_{T}$CYC$_{1}$*	HIS3	HIS3	This study
pENZ004-mKate2	*TDH3p-mKATE2*	TRP1	ChrX site II	This study
pENZ030	*TDH3p-ADH1t*	LEU2	HO	This study
pENZ030-UbiC	*TDH3p- EcUbiC-ADH1t*	LEU2	HO	This study

Supplementary Table 3. Codon optimized sequences used in this study.

Gene Name	Sequence
**HbaR**	ACCGGATTGAGCTTTCAATTGTTCAACTCTTTGGACCAAGTGATGCATATGTTGGCAAATAGAAGC<br>AATCATTGGAACGGAGATATCTGGGTTAGCTCTAATGCATCTAACCAAATGATCAGCTGGAATTCT<br>AGCAACTCTAGCATCGGTAACTGCTTCAGCATTAGCCAAATATCTATGACCAGTCAAAGCAGCAG<br>CTTCAGCAAAAGAACCACCTCTAGACATAATTTCGATAACGGCTTCATCACCAGCCAAATTAGATC<br>TGAACAACTTGACCCAACCACCAACAACTATGAAAAAAGCTGTAGCTGGTTCATCTTGTCTGGCAA<br>TAGATTCTCTGGCTCTAACAGAAATAGCAGTAGCTGGAGCAATCATATGAGCAACAGTTTCAGCCT<br>TCAAACCTCTGAAAACAGCAATTCTGGTGATAATTTGCAAGTCACCACCAGTCAATTTAGAAGC
**UbiC**	ATGTCTCACCCTGCACTTACACAGTTGAGGGCTTTGAGGTACTGTAAAGAAATCCCTGCTTTGGAC<br>CCGCAGTTGTTAGACTGGTTGCTGCTGGAAGACAGCATGACGAAAAGATTTGAGCAGCAAGGGAA<br>AACAGTCTCAGTAACAATGATAAGGGAAGGTTTTGTGGAACAAAATGAAATCCCTGAGGAACTTC<br>CATTACTTCCCAAGGAGTCTCGTTACTGGCTTCGTGAGATTCTTCTGTGTGCCGACGGAGAGCCGT<br>GGCTTGCAGGCCGTACTGTGGTACCAGTAAGTACATTATCTGGCCCGGAGCTGGCACTTCAAAAGC<br>TAGGAAAGACACCCTTAGGCAGGTATCTGTTTACAAGTAGCACATTGACCAGAGACTTTATAGAG<br>ATTGGAAGGGACGCTGGTTTGTGGGGGCGTAGGTCTAGACTGCGTCTATCCGGCAAACCCCTACTT<br>TTAACTGAACTTTTCCTACCAGCAAGTCCTTTGTAA
**Aro4 K229L**	ATGTCAGAAAGTCCAATGTTCGCTGCCAATGGCATGCCAAAAGTGAACCAAGGCGCCGAAGAGGA<br>CGTGAGGATTCTAGGGTACGATCCATTGGCTTCACCTGCCTTGTTGCAAGTACAGATACCAGCGAC<br>TCCAACGAGCTTGGAAACGGCAAAGAGGGGTAGGAGAGAAGCTATAGACATAATTACGGGAAAA<br>GACGACAGGGTGCTGGTGATCGTTGGCCCCTGCAGTATCCATGACCTAGAGGCTGCACAGGAGTA<br>CGCTCTGAGGCTTAAGAAGCTGTCCGATGAATTAAAGGGCGATTTATCCATAATTATGAGGGCGTA<br>TCTGGAAAAGCCGCGTACTACCGTTGGCTGGAAGGGTCTAATCAATGATCCGGATGTTAATAATAC<br>CTTTAACATCAATAAAGGACTACAATCCGCTAGGCAGCTGTTCGTGAATTTGACGAACATTGGCCT<br>TCCTATAGGCTCCGAAATGTTAGATACAATCTCTCCTCAGTACCTGGCAGACTTAGTATCATTTGG<br>AGCAATCGGGGCAAGAACCACGGAATCACAACTGCACAGGGAACTTGCGTCAGGTCTTTCTTTCC<br>CGGTAGGCTTTAAAAATGGCACGGATGGCACACTAAATGTTGCGGTAGACGCATGCCAAGCTGCA<br>GCGCATTCTCATCACTTCATGGGTGTAACCTTGCATGGCGTGGCAGCAATTACAACAACAAAGGGC<br>AACGAACATTGTTTCGTTATACTTCGTGGCGGGAAAAAGGGTACGAACTACGACGCGAAATCTGT<br>AGCAGAGGCAAAGGCACAATTACCTGCTGGGTCCAATGGGCTTATGATTGACTACAGTCACGGCA<br>ATTCCAATAAAGATTTCAGGAACCAACCTAAGGTAAACGATGTGGTGTGCGAGCAAATAGCAAAT<br>GGTGAGAACGCAATTACGGGAGTTATGATCGAATCTAACATCAATGAGGGAAACCAGGGGATCCC<br>TGCTGAGGGGAAGGCTGGACTGAAATATGGAGTTAGCATAACAGATGCCTGTATAGGTTGGGAAA<br>CGACAGAGGATGTGCTTAGAAAGTTAGCCGCCGCCGTACGTCAAAGGAGGGAGGTGAATAAGAA<br>A

Supplementary Table 4. pKa of all the compounds tested in this study.

Metabolite	pKa	Source
**HBA**	4.54	https://pubchem.ncbi.nlm.nih.gov/compound/4-hydroxybenzoic_acid#section=LogP
**3HBA**	4.08	https://www.chemicalbook.com/ProductMSDSDetailCB4854760_EN.htm
**2HBA**	2.97	https://pubchem.ncbi.nlm.nih.gov/compound/338
**pABA**	4.77	https://www.drugbank.ca/drugs/DB02362
**3NBA**	4.78	https://www.chemicalbook.com/ProductList_En.aspx?kwd=3-amino%20benzoic%20acid
**2NBA**	4.89	https://www.drugbank.ca/drugs/DB04166
**3,4DHBA**	4.26	https://pubchem.ncbi.nlm.nih.gov/compound/72#section=Solubility
**2,5HBA**	3.01	https://www.chemicalbook.com/ProductMSDSDetailCB5853669_EN.htm
**Benzoic acid**	4.19	https://pubchem.ncbi.nlm.nih.gov/compound/243#section=pH
**Ferulic acid**	4.42	https://pubchem.ncbi.nlm.nih.gov/compound/445858#section=LogP
**Homovanillic acid**	4.39	https://www.ebi.ac.uk/chembl/compound/inspect/CHEMBL1562
**Nicotinic acid**	4.75	https://pubchem.ncbi.nlm.nih.gov/compound/nicotinic_acid#section=Top
**Acetyl Salicylic Acid**	3.49	https://pubchem.ncbi.nlm.nih.gov/compound/aspirin#section=Decomposition
**Vanillic acid**	4.51	https://pubchem.ncbi.nlm.nih.gov/compound/8468#section=LogP

Supplementary Table 5. Specific growth rates of derivatives strains ySCC185-F in the HBA batch production cultures.

**Metabolite**	**μ^{max} (h^{-1})**
Control	0.21±0.05
UbiC	0.20±0.02

SUPPLEMENTARY FIGURES

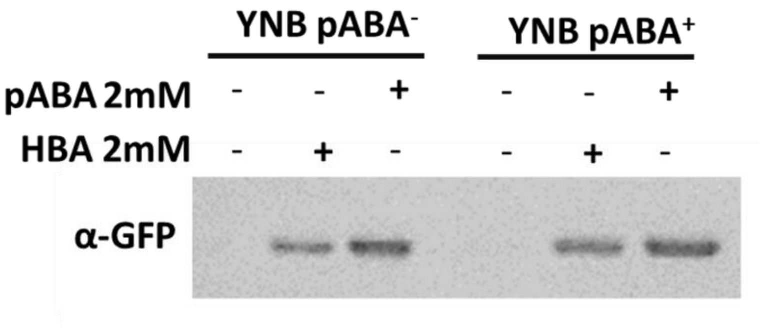

Supplementary Figure 1. Western blot against mCitrine expressed in the strain ySCC185-F under different culture conditions. The mCitrine was detected using the αGFP antibody.

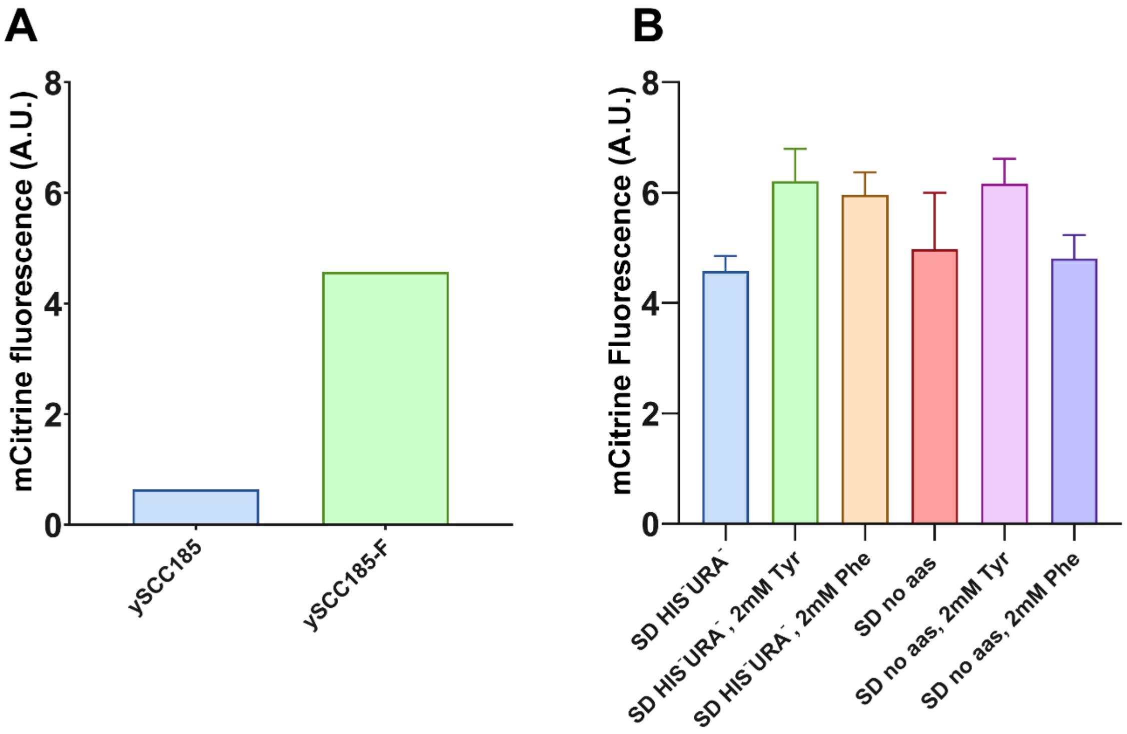

Supplementary Figure 2. mCitrine fluorescence in different control conditions. **A)** mCitrine was measured in the strain ySCC185 not carrying sHbaR. **B)** mCitrine fluorescence in different culture conditions, SD with CSM His$^-$ and Ura$^-$ and SD without any amino acids. Tyrosine or phenylalanine were also supplemented for both culture conditions.

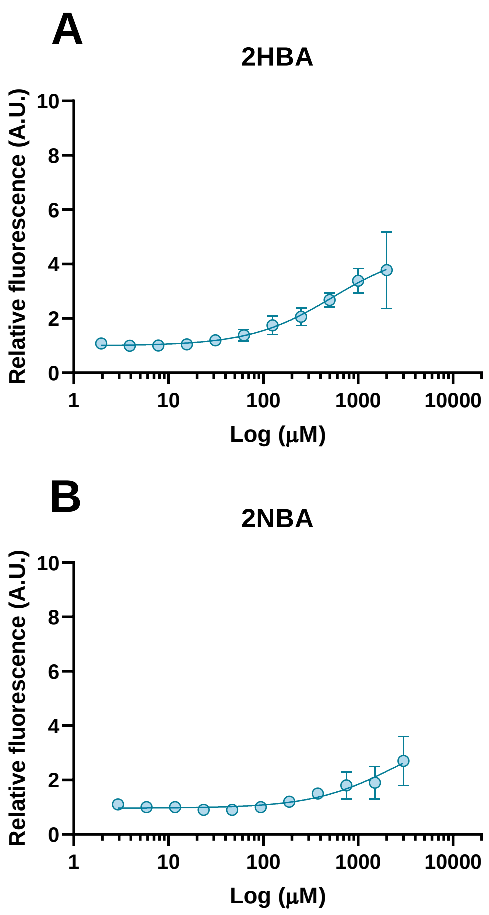

Supplementary Figure 3. Dose response curves obtained with 2HBA and 2NBA of sHbaR expressed in *S. cerevisiae*. mCitrine fluorescence was measured by flow cytometry. In these graphs the relative fluorescence of mCitrine of cells grown at different concentration of A) 2HBA and B) 2NBA compared with the control condition are presented. Experiments were carried out in three biological replicates.

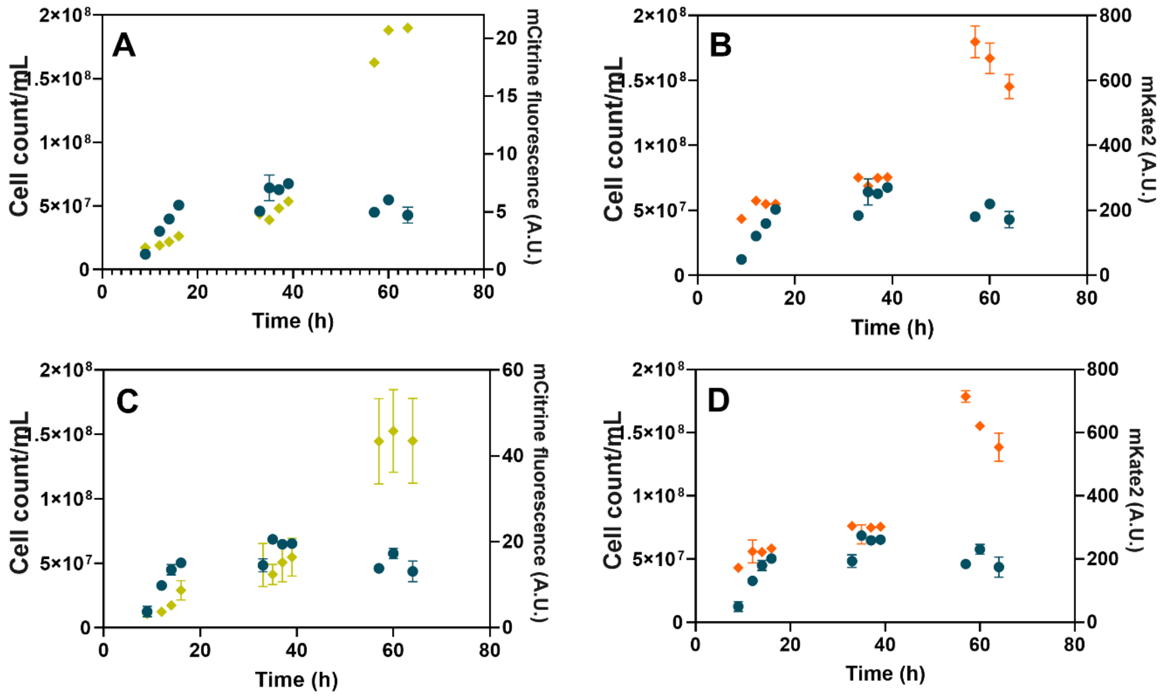

Supplementary Figure 4. Comparison of cell count and mCitrine fluorescence (**A** and **B**) or mKate2 fluorescence (**B** and **D**) of the strains ySCC185-30 (**A** and **B**) and the HBA producing strain ySCC185-UbiC (**C** and **D**) during 65 hours of cultures in synthetic medium. Experiments were carried out with three biological replicates.

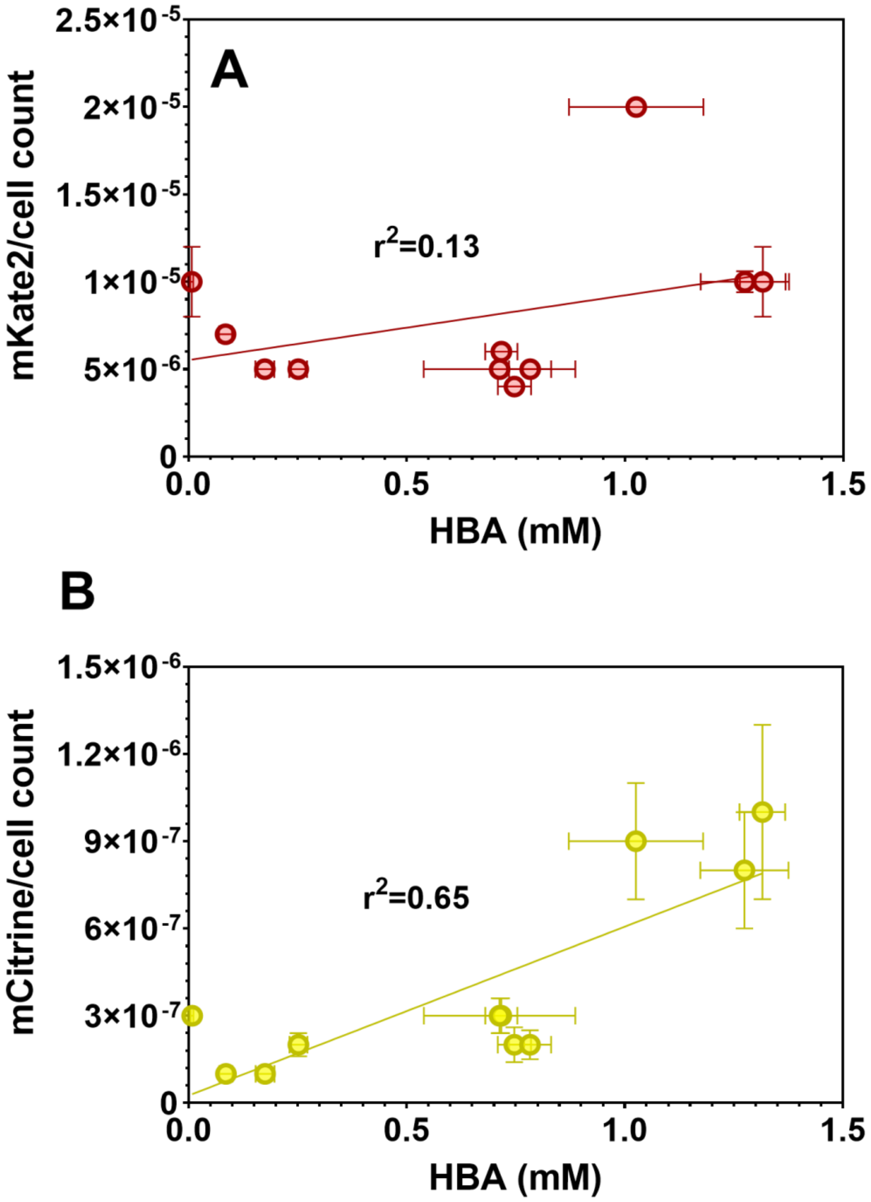

Supplementary Figure 5. Correlation between HBA production of the strain ySCC185-UbiC compared with the relative values of **A)** mKATE2 fluorescence/cell concentration and **B)** mCitrine fluorescence/cell concentration. Experiments were carried out with three biological replicates.
